# Racial and Ethnic Disparities in Fathers' Food Parenting Practices and Children's Diets

**DOI:** 10.1111/cdev.70001

**Published:** 2025-06-09

**Authors:** Yilin Wang, Brian K. Lo, In Young Park, Katherine W. Bauer, Kirsten K. Davison, Jess Haines, Rebekah Levine Coley

**Affiliations:** ^1^ Department of Counseling, Developmental, and Educational Psychology Boston College Chestnut Hill Massachusetts USA; ^2^ Department of Family Relations & Applied Nutrition University of Guelph Guelph Canada; ^3^ Department of Social Welfare Chungnam National University Daejeon Republic of Korea; ^4^ School of Public Health University of Michigan Ann Arbor Michigan USA; ^5^ School of Social Work Boston College Chestnut Hill Massachusetts USA

**Keywords:** father, food parenting, race and ethnicity

## Abstract

Racial and ethnic disparities in children's diets are prevalent. Little is known about how fathers' food parenting practices may contribute to these disparities. We examined racial and ethnic variations in food parenting practices and their associations with 2–6‐year‐old children's diets in a cross‐sectional sample of U.S. fathers surveyed in 2021–2023 (*N* = 1015; 16% Asian, 9% Black, 6% Hispanic, 70% White; *M*_age_ = 37 years) using path analysis. Fathers' food parenting practices were significantly associated with children's diets, yet little evidence emerged that fathers' food parenting practices explained racial and ethnic disparities in children's diets. These findings suggest the potential importance of structural constraints on healthy eating (e.g., access to healthy food) among minoritized children beyond fathers' food parenting practices.

Racial and ethnic disparities in young children's dietary intake are prevalent in the United States. Recent evidence shows that compared with White children, Hispanic and Black children consume fewer fruits and vegetables and more sugar‐sweetened beverages (Demmer et al. 2018; Hamner 2023). It is important to understand factors that contribute to these dietary differences, as young children's dietary intake contributes to their healthy growth, weight, and cognitive development (Clark et al. 2020).

One important family‐level factor that has been consistently found relevant to children's diets is food parenting practices (e.g., Chen et al. 2021), which refer to the feeding strategies used by parents to influence their children's nutrition‐related behavior (Musher‐Eizenman and Holub 2007). Recent scholarship on food parenting practices has identified three fundamental domains of such practices, including coercive control, structure, and autonomy support (Vaughn et al. 2016). The vast majority of extant literature on food parenting practices has focused on mothers or parents in general, while fathers have been underrepresented in the food parenting literature, despite their critical roles as nuclear family members and increasing involvement in child feeding (Davison et al. 2020). Moreover, there has been a limited focus on the food parenting practices of fathers of color, leaving the role of food parenting practices in explaining racial and ethnic disparities in children's diets understudied (Davison et al. 2020). To address this gap, our study examined racial and ethnic differences in fathers' food parenting practices and their associations with children's diets in a racially and ethnically diverse sample of preschool‐aged children and fathers living in the United States.

## 1 Fathers' Food Parenting Practices and Children's Diets

The bioecological systems theory emphasizes the family as one of the most important contexts for child development (Bronfenbrenner and Morris 2006), laying the foundation for theoretical models focusing specifically on child health outcomes to propose food parenting practices as key predictors of children's dietary intake (Davison and Birch 2001). Recent scholarship on food parenting practices has identified three fundamental domains, including coercive control, structure, and autonomy support (Vaughn et al. 2016). These domains are rooted in parenting styles characterized by involvement, structure, and autonomy support (Grolnick and Lerner 2023). Specific to food parenting (Vaughn et al. 2016), coercive control refers to food parenting practices that aim to take excessive control of children's food intake, such as using food to regulate children's emotions or reward children. Structure refers to parental practices seeking to organize their children's food environment, such as monitoring their children's food intake and food availability at home. Autonomy support refers to parenting practices that aim to encourage children's independence in their dietary behaviors, such as encouraging children to eat healthy foods and be involved in preparing family meals. Evidence—nearly all from studies focused on mothers—has shown that coercive control is counterproductive to promoting healthy diets for children, while structure and autonomy support are more beneficial for children eating healthily (Chen et al. 2021).

Despite a few qualitative studies (e.g., Roche et al. 2015), relatively few studies have directly assessed fathers' food parenting practices (Moura and Philippe 2023). Previous food parenting literature primarily examined parents collectively (e.g., Inhulsen et al. 2017) or used mothers' reports of fathers' food parenting practices (e.g., Lora et al. 2017). However, this may lead to less accurate reports of fathers' practices, as they tend to use different strategies than mothers, such as less structure‐based practices (Pratt et al. 2019).

In addition, research has not assessed links between the three fundamental domains of food parenting practices among fathers and children's diets (Davison et al. 2020). A limited number of recent studies on fathers' food parenting practices have found associations between specific practices and children's diets, such as using food as a reward, using food to calm children, and eating with children in restaurants (Lora et al. 2016; Parada et al. 2016). However, this focus on individual food parenting practices rather than higher‐order food parenting domains limits the ability to compare across studies regarding food parenting. By incorporating the theory of parenting styles to conceptualize domains of food parenting practices, research may gain greater insight into the underlying reasons for and implications of fathers' food parenting practices. One recent study on fathers' food parenting practices assessed in conceptual domains found that fathers reported higher use of coercive control compared to mothers, but did not link fathers' food parenting with children's diets (Pratt et al. 2019).

## 2 Fathers' Race and Ethnicity and Food Parenting Practices

As prior theoretical models on child health outcomes propose, the relation between food parenting practices and child diets is embedded in social and cultural contexts, such as racial and ethnic norms (Davison and Birch 2001). However, this argument has not been widely explored in empirical studies, as most research on fathers' food parenting practices has relied on small samples of White fathers (Davison et al. 2020). There has been a growing, though still limited, body of studies focusing on the feeding strategies of fathers from specific racial and ethnic groups, particularly Hispanic fathers (Lora et al. 2017; Parada et al. 2016). However, fewer studies have included diverse groups of fathers of color to examine variations or consistencies in food parenting practices across racial and ethnic groups (for an exception, see Lora et al. 2016, which compared Black and Hispanic fathers' food parenting practices).

Although the model proposed by Davison and colleagues did not specify how race and ethnicity may intersect with food parenting practices to influence child dietary intake (Davison and Birch 2001), current theoretical scholarship suggests two potential roles: On one hand, fathers' race and ethnicity, as a proxy of cultural norms, economic opportunities, and social stressors (García Coll et al. 1996), may drive differences in food parenting practices, in turn leading to differences in children's diets. For example, although not specific to fathers, Polfuss and Frenn (2012) found that Black parents of school‐aged children adopted more controlling food parenting practices compared to White parents. Such differences might, in turn, lead to differences in children's diets, although extant research on fathers has not directly addressed this hypothesis.

On the other hand, fathers' race and ethnicity may moderate the relation between their food parenting practices and children's diets. For example, Lansford (2022) argues that parenting behavior may have stronger effects when it aligns with cultural norms. This argument is supported by empirical studies on physical discipline (Lansford et al. 2004), but has received less attention in relation to food parenting practices. Only one study, to our knowledge, has assessed racial and ethnic differences in associations between fathers' food parenting practices and preschool‐aged children's diets, in particular, children's intake of sugar‐sweetened beverages (Lora et al. 2016). This study found a significant positive association between using food to calm children and children's sugar‐sweetened beverage intake among Hispanic fathers, but not among Black fathers. Although this study takes an initial step toward understanding the food parenting practices of racial and ethnic minority fathers, more research is needed to assess a broader array of fathers' food parenting practices and children's dietary behaviors across racial and ethnic groups.

## 3 Current Study

**FIGURE 1:**
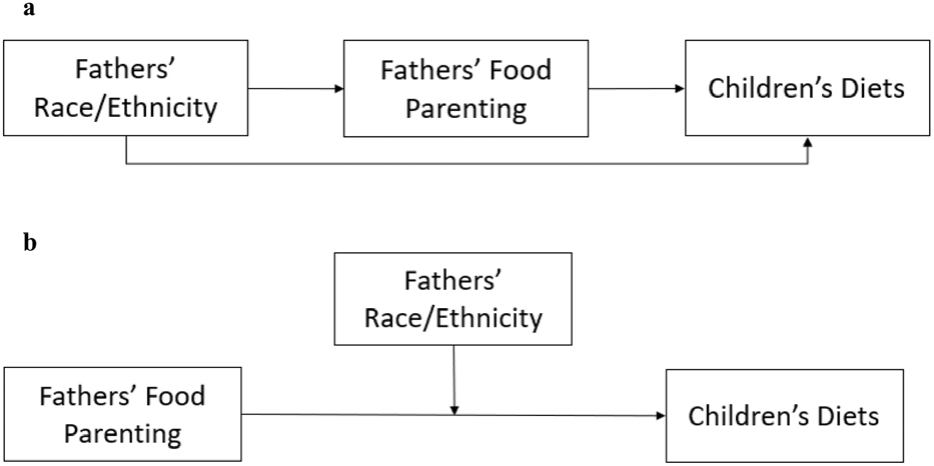
(a) Mediation Conceptual Model: fathers' food parenting practices as mediators between fathers' race/ethnicity and children's diets. (b) Moderation Conceptual Model: fathers' race and ethnicity as moderators of the relation between fathers' food parenting practices and children's diets.

Our study aims to address these gaps by exploring the association between three domains of fathers' food parenting practices and four components of children's diets, and how this association varies across four racial and ethnic groups: Non‐Hispanic Asian (Asian), Non‐Hispanic Black (Black), Hispanic (of any race), and Non‐Hispanic White (White). Based on the theoretical proposals discussed above, our study asks two research questions. Our first question investigates whether fathers' food parenting practices mediate the association between fathers' race and ethnicity and children's diets, expecting that fathers' food parenting practices will vary across racial and ethnic groups and thus lead to differences in children's diets (Figure 1a). Our second question examines whether fathers' race and ethnicity moderate the association between their food parenting practices and children's diets, hypothesizing that this association will vary across racial and ethnic groups (Figure 1b). For both questions, the limited prior empirical and theoretical evidence inhibited the development of more specific and directional expectations regarding racial and ethnic differences, making these hypotheses exploratory. By contrasting these two models, we seek to delve into racial and ethnic disparities in children's diets, aiming to delineate racial and ethnic variation in the uses and implications of fathers' food parenting practices within the domains of coercive control, structure, and autonomy support.

## 2 Method

### 2.1 Sample and Procedures

The cross‐sectional sample for our study (*N* = 1272 fathers) was drawn from the baseline survey of the Fathers & Families Study, a longitudinal study that investigates the role of fathers in promoting child health. Participants were initially recruited between July 2021 and June 2022 from the existing cohort of the Growing Up Today (GUTS) study, a longitudinal study following more than 27,000 children of nurses since early adolescence (https://gutsweb.org/about‐guts/). To increase the racial and ethnic diversity of the sample, a second phase of recruitment was conducted from June 2022 to June 2023 in collaboration with Michigan Medicine, the health system of the University of Michigan (MM). During both phases, fathers were recruited through email and mail. Fathers who identified as biological, social, or adoptive fathers of children aged 1–6 years and who lived with the child (ren) for at least 50% of the time in the United States were eligible for the study. Those who completed the screener and met the eligibility criteria were enrolled in the Fathers & Families Study, including 750 fathers from GUTS and 522 from MM. Although participants reside throughout the US, the Fathers & Families Study is not a nationally representative sample. Participants completed an online survey administered via REDCap. Procedures were approved by Boston College and met institutional guidelines for the protection of human subjects.

Of the fathers who participated in the baseline survey, we focused our analytic sample on fathers who were Asian, Black, Hispanic (of any race), and White. Fathers from other racial and ethnic groups (e.g., Native Hawaiians and other Pacific Islanders) were excluded due to their limited numbers (*n* = 38). We also excluded fathers whose children were under age 2 (*n* = 194) because food parenting practices with infants and toddlers could be significantly different from those with preschool‐aged children (Russell et al. 2018). These exclusions, along with removing cases with missing data due to very low levels of missingness (ranging from 1.25% to 2.12% across analytic variables), led to an analytic sample of 1015 fathers.

### 2.2 Measures

#### 2.2.1 Fathers' Food Parenting Practices

Fathers reported their food parenting practices using 16 questions drawn from the Comprehensive Feeding Practice Questionnaire (CFPQ; Musher‐Eizenman and Holub 2007). The adapted survey includes one item each from the modeling and involvement subscales and two items each from the monitoring, child control, emotion regulation, encouraging balance and variety, environment, food as reward, and restriction for health subscales. All questions were answered on a “strongly disagree” to “strongly agree” four‐point scale. In a previous study (Lo et al. 2025), confirmatory factor analyses were used to confirm the presence of the three higher‐order parenting domains from 11 items: coercive control, structure, and autonomy support. Coercive control was measured using four items assessing paternal control of their children's dietary intake (e.g., “I offer sweets to my child as a reward for good behavior”). Structure was measured using five items assessing fathers' efforts to organize children's food environment (e.g., “I keep a lot of snack food (potato chips, Doritos, cheese puffs)”). Autonomy support was measured using two questions assessing how fathers encouraged balance and variety in children's diets (e.g., “I tell my child that healthy food tastes good”). We combined food parenting practice items into composite scores for each domain by taking the mean of scale items. The internal reliability of these scales was modest both for the overall sample (coercive control: *α* = 0.60; structure: *α* = 0.69; autonomy support: *α* = 0.59), as well as for Asian (coercive control: *α* = 0.61; structure: *α* = 0.67; autonomy support: *α* = 0.50), Black (coercive control: *α* = 0.48; structure: *α* = 0.61; autonomy support: *α* = 0.62), Hispanic (coercive control: *α* = 0.58; structure: *α* = 0.58; autonomy support: *α* = 0.62), and White (coercive control: *α* = 0.61; structure: *α* = 0.71; autonomy support: *α* = 0.62) fathers, likely influenced by the small number of items in each domain.

#### 2.2.2 Child Diet

Fathers reported on children's consumption of fruits, vegetables, sugar‐sweetened beverages (including soda, sweetened drinks, and flavored milk), and fast food, including “meals and snacks eaten at home, at school, at restaurants, and anywhere else” over the past 30 days. Items were drawn from the National Health and Nutrition Examination Survey (NHANES) Dietary Screener Questionnaire (National Center for Health Statistics 2023) and answered on a seven‐point scale ranging from “Never” to “5 or more times a day.”

We dichotomized responses for each type of food as meeting or not meeting dietary recommendations. All dietary cut‐offs were informed by the 2020–2025 Dietary Guidelines for Americans (DGA) (U.S. Department of Agriculture and U.S. Department of Health and Human Services 2020) and prior research. The DGA recommends daily servings of fruits and vegetables based on portion sizes for children aged 2–8. Extrapolating from the guidelines and following the coding approach used in prior studies (Gago et al. 2023), we coded children's consumption of fruits and vegetables as meeting dietary guidelines if they consumed each type of food two or more times per day.

Similarly, the DGA recommends limiting the consumption of sugar‐sweetened beverages but does not offer specific recommendations for fast food and the frequency of consuming sugar‐sweetened beverages. Building on the guidelines and informed by dietary cut‐offs clinically relevant to obesity risk from prior studies (Davis et al. 2007; Ludwig et al. 2001), we coded children's consumption of sugar‐sweetened beverages and fast food as meeting dietary guidelines if each type of food was consumed less than once per week. In alternate models, we also assessed continuous measures of consumption, assessed as times per week, for each of the four food types.

#### 2.2.3 Covariates

Child‐level covariates included child age (in years) and gender (0 = female, 1 = male), which were found to be associated with children's diet and food parenting in prior studies (Inhulsen et al. 2017). Informed by prior research (Benmarhnia et al. 2021), we assessed racial and ethnic differences unadjusted for associated socioeconomic status and family structure, which have been found to vary systematically across racial and ethnic groups in the U.S. (Williams et al. 2016) and may be on the causal path from race/ethnicity to parenting and to child diet. As such, the inclusion of such covariates would mask the raw racial and ethnic differences in food parenting and children's diets that we sought to explore.

### 2.3 Data Analysis

We addressed our research questions using path analysis conducted in R version 3.4.4 using the *lavaan* package (Rosseel 2012). First, descriptive analysis of racial and ethnic differences in children's diets and food parenting and the bivariate association between these key variables were assessed. A mediation model was then estimated to test the direct effect of fathers' race and ethnicity on children's diets and indirect effects via food parenting practices. The four child dietary outcomes were regressed in probit models on three food parenting practices composites and three racial and ethnic indicators. The three food parenting practices composites were regressed in linear models on three racial and ethnic indicators. The covariates (child age and gender) were included as predictors of both the mediators and outcomes. Covariances between pairs of food parenting practices composites and between pairs of child diet variables were also estimated, eliminating nonsignificant covariances between fruit and fast food intake and between vegetable and sugar‐sweetened beverage intake to improve model fit. Direct, indirect, and total effects were estimated.

A multi‐group model was then used to test the moderating role of race and ethnicity on the relation between fathers' food parenting practices composites and children's diets, using fathers' race and ethnicity as the grouping variable. The model comparison approach was used to examine significant differences in paths across racial and ethnic groups. To compare models, we conducted the likelihood ratio (LR) test, which is obtained by estimating two nested models with and without constraints (Svetina et al. 2020). Specifically, we started by constraining all main paths (i.e., paths between food parenting practices composites and children's diets) to be equal across all groups. LR results not showing a significant degradation in the model fit of the constrained model indicate no significant differences across racial and ethnic groups. LR results showing that the model fit of the constrained model significantly degraded compared with the unrestrained one suggest variability across groups, which can be further assessed by releasing the constraint of each path sequentially and allowing it to vary across groups, determining significant variation by improvement in model fit.

## 3 Results

### 3.1 Descriptive Results

Table 1 provides a summary of the sample's demographic characteristics. In the analytic sample (*N* = 1015), 16% of fathers were Asian, 9% Black, 6% Hispanic, and 70% White. Fathers were 37 years old on average, mostly married (96%) and employed full‐time (92%). Although the sample was socioeconomically advantaged overall, disparities across racial and ethnic groups emerged similar to national patterns (Williams et al. 2016). The majority of fathers in the sample held a college degree (84%), with the highest proportion among Asian fathers (96%) and the lowest among Black fathers (46%). The average annual household income for the sample was $130,380, with Asian fathers reporting the highest average income ($150,120) and Black fathers the lowest ($84,700). Children of fathers were 3.8 years of age on average, and just over half were male (54%).

**TABLE 1:** Sample demographics.

Characteristics	Full sample	Asian	Black	Hispanic	White
*N* Percentage	*N* = 1015	*n* = 159 (15.67%)	*n* = 90 (8.87%)	*n* = 59 (5.81%)	*n* = 707 (69.66%)
Fathers					
Father age (years)	36.54 (4.15)	38.04^h,w^ (4.08)	37.04 (7.48)	36.39^a^ (5.47)	36.15^a^ (3.29)
College degree	84.49%	95.60%^b,h,w^	45.56%^a,h,w^	66.67%^a,b,w^	88.39%^a,b,h^
Household income ($10,000)	13.38 (8.31)	15.12^b,h^ (8.68)	8.47^a,w^ (6.86)	10.41^a,w^ (7.57)	13.85^b,h^ (8.18)
Employed	91.92%	94.94%^b^	78.82%^h,b,w^	91.38%^b^	92.88%^b^
Married	96.33%	98.69%^b,h^	76.54%^a,h,w^	89.47%^a,b,w^	98.70%^b,h^
Children					
Child is male	53.50%	52.83%	54.44%	52.54%	53.61%
Child age (years)	3.85 (1.32)	4.31^h,w^ (1.39)	4.12^w^ (1.46)	3.72^a^ (1.21)	3.72^a,b^ (1.27)

*Note:* Mean (SD) or percent presented. Superscripts indicate significant differences (*p* < 0.05) from the labeled group: a, Asian; b, Black; h, Hispanic; w, White.

Table 2 presents descriptive data on our primary variables of interest. Significant differences were apparent in children's diets across racial and ethnic groups. Specifically, children of Black fathers were least likely to meet recommendations for all four diet outcomes: they were significantly less likely than children of White fathers to meet recommendations for all dietary intake; less likely to meet the recommendation for sugar‐sweetened beverages and fast food consumption in comparison to children of Asian fathers; and less likely to meet the recommendation for fruit intake compared with peers of Hispanic fathers. In addition, children of Asian fathers were less likely to meet the recommendation for fruit consumption than those of White fathers. In contrast, bivariate comparisons showed few racial and ethnic differences in fathers' food parenting practices. The only significant difference was that Asian fathers reported significantly lower autonomy support than their Black and White peers.

**TABLE 2:** Descriptive statistics of child diets and food parenting practices by father race/ethnicity.

	Full sample	Asian	Black	Hispanic	White
Child diets: % meet recommendation					
Fruit	47.64	37.74^w^	30.4^h,w^	47.46^b^	52.1^a,b^
Vegetables	23.85	23.27	15.05^w^	15.25	25.85^b^
SSBs	48.75	51.23^b^	37.11^a,w^	45.00	50.07^b^
Fast food	64.28	70.4^b^	47.31^a,w^	59.32	65.54^b^
Food parenting practices: Mean (SD)					
Coercive control	2.05 (0.48)	2.08 (0.53)	2.10 (0.48)	2.02 (0.48)	2.04 (0.47)
Structure	3.05 (0.45)	3.03 (0.45)	3.07 (0.44)	3.07 (0.43)	3.05 (0.45)
Autonomy support	3.46 (0.49)	3.34 (0.48)^b,w^	3.54 (0.47)^a^	3.48 (0.47)	3.48 (0.49)^a^

*Note:* Superscripts indicate significant differences (*p* < 0.05) from the labeled group: a, Asian; b, Black, h, Hispanic, w, White.

Abbreviation: SSBs, sugar‐sweetened beverages.

We also examined correlations between fathers' food parenting practices and children's diets (Table 3). Consistent with another study using Fathers & Families Study data (Lo et al. 2025), we found statistically significant associations between all food parenting and child diet measures except for the association between autonomy support and the consumption of sugar‐sweetened beverages. That is, higher coercive control was associated with a lower likelihood of meeting dietary guidelines for all four food types, while higher structure and autonomy support were associated with a greater likelihood of meeting dietary guidelines.

**TABLE 3:** Correlation between fathers' food parenting and children's diets.

	Coercive control	Structure	Autonomy support	Fruit	Vegetables	SSBs	Fast food
Control	1						
Structure	−0.29***	1					
Support	−0.15***	0.39***	1				
Fruit	−0.15***	0.15***	0.14***	1			
Vegetables	−0.17***	0.24***	0.21***	0.37***	1		
SSBs	−0.22***	0.24***	0.05	0.13***	0.11**	1	
Fast food	−0.18***	0.27***	0.12***	0.11***	0.15***	0.28***	1

*Note:* Pearson correlations are presented among three composites of fathers' food parenting practices. Point‐biserial correlations are presented between fathers' food parenting practices and child diets, and phi coefficients among child diet variables.

Abbreviation: SSBs, sugar‐sweetened beverages.

***p < 0.001, **p < 0.01.

### 3.2 Mediation Results

**FIGURE 2:**
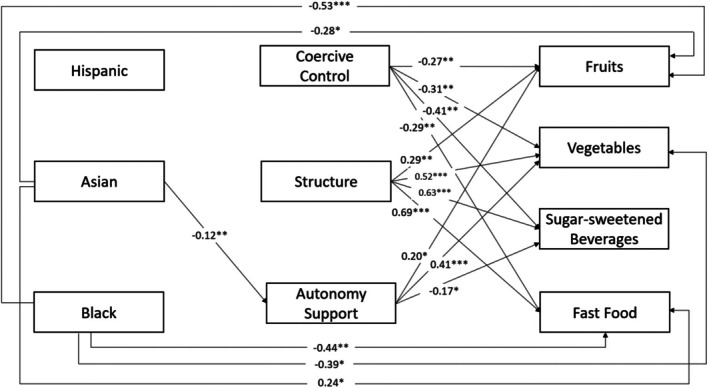
Mediation Path Analysis linking fathers' race and ethnicity to children's diets through food parenting practices. Significant paths (*p* < 0.05) are presented in the diagram. Unstandardized coefficients are presented for each significant path. Models control for child age and gender. White is the omitted race group.

Table 4 presents unstandardized regression coefficients and standard errors for the primary paths and covariate paths in the mediation model. The largest racial group of White fathers was the omitted group; post hoc analyses tested significant differences between other racial and ethnic group pairs. Figure 2 presents the results for the statistically significant paths and associated unstandardized coefficients (covariates and differences between other racial and ethnic groups not presented). We also present direct, indirect, and total effect estimates in Table 5. The adjusted model fit the data well (*χ* ^2^[2] = 3.086; CFI = 0.999; RMSEA = 0.023, 90% CI = 0.000–0.071; SRMR = 0.016).

**TABLE 4:** Mediational Path Model results linking fathers' race and ethnicity to child diets through food parenting practices.

Variables	Probit regression on child diet				Linear regression on food parenting		
	Fruit	Veg	SSBs	Fast food	Control	Structure	Support
	*b* (S.E.)	*b* (S.E.)	*b* (S.E.)	*b* (S.E.)	*b* (S.E.)	*b* (S.E.)	*b* (S.E.)
Father race/ethnicity → Father food parenting practices							
Asian					0.01 (0.04)	−0.01 (0.04)	−0.12**^b^ (0.04)
Black					0.04 (0.05)	0.03 (0.05)	0.07^a^ (0.05)
Hispanic					−0.03 (0.07)	0.01 (0.06)	−0.01 (0.06)
Father food parenting practices → child diet							
Coercive control	−0.27** (0.09)	−0.31** (0.09)	−0.41*** (0.09)	−0.29** (0.09)			
Structure	0.29** (0.10)	0.52*** (0.10)	0.63*** (0.09)	0.69*** (0.10)			
Autonomy support	0.20* (0.09)	0.41*** (0.10)	−0.17* (0.09)	0.04 (0.09)			
Father race/ethnicity → child diet							
Asian	−0.28* (0.11)	−0.02^b^ (0.12)	0.15^b^ (0.11)	0.24*^b,h^ (0.12)			
Black	−0.53*** (0.15)	−0.39*^a^ (0.17)	−0.19^a^ (0.13)	−0.44**^a^ (0.14)			
Hispanic	−0.13 (0.18)	−0.39^+^ (0.19)	−0.16 (0.18)	−0.19^a^ (0.17)			
Age	−0.07* (0.03)	0.02 (0.03)	−0.19*** (0.03)	−0.11*** (0.03)	0.05*** (0.01)	−0.01 (0.01)	−0.02+ (0.01)
Male	0.12 (0.08)	0.02 (0.08)	−0.10 (0.08)	−0.12 (0.08)	−0.01 (0.03)	−0.01 (0.03)	−0.07* (0.03)

*Note:* White is the reference group. Significant differences between other groups are indicated with superscripts (a, Asian; b, Black; h Hispanic).

Abbreviations: SSBs, sugar‐sweetened beverages; Veg, vegetables.

***p < 0.001, **p < 0.01, *p < 0.05, +*p* < 0.10.

**TABLE 5:** Mediational Path Model indirect, direct, and total effects.

Indirect effects	Fruit	Vegetables	SSBs	Fast food
	*b* (S.E.)	*b* (S.E.)	*b* (S.E.)	*b* (S.E.)
Asian				
Via coercive control	0.00 (0.01)	0.00 (0.01)	0.00 (0.02)	0.00 (0.01)
Via structure	0.00 (0.01)	0.00 (0.02)	−0.01 (0.03)	−0.01 (0.03)
Via autonomy support	−0.03 (0.02)	−0.05*^b^ (0.02)	0.02^b^ (0.01)	0.00 (0.01)
Direct effect	−0.28* (0.11)	−0.02 (0.12)	0.15 (0.11)	0.24* (0.11)
Total effect	−0.32** (0.11)	−0.07 (0.12)	0.16^b^ (0.11)	0.23^+b^ (0.12)
Black				
Via coercive control	−0.01 (0.02)	−0.01 (0.02)	−0.02 (0.02)	−0.01 (0.02)
Via structure	0.01 (0.01)	0.01 (0.02)	0.02 (0.03)	0.02 (0.03)
Via autonomy support	0.01 (0.01)	0.03^a^ (0.02)	−0.01^a^ (0.01)	0.00 (0.01)
Direct effect	−0.53*** (0.15)	−0.39* (0.17)	−0.19 (0.13)	−0.44**^a^ (0.14)
Total effect	−0.51*** (0.15)	−0.36* (0.17)	−0.21^a^ (0.14)	−0.44**^a^ (0.14)
Hispanic				
Via coercive control	0.01 (0.02)	0.01 (0.02)	0.01 (0.03)	0.01 (0.02)
Via structure	0.00 (0.02)	0.01 (0.03)	0.01 (0.04)	0.01 (0.04)
Via autonomy support	0.00 (0.01)	0.00 (0.03)	0.00 (0.01)	0.00 (0.01)
Direct effect	−0.13 (0.18)	−0.39^+^ (0.19)	−0.14 (0.18)	−0.19 (0.16)
Total effect	−0.12 (0.18)	−0.38^+^ (0.20)	−0.13 (0.18)	−0.17 (0.19)

*Note:* White is the reference group. Significant differences between other groups are indicated with superscripts (a Asian, b Black).

Abbreviation: SSBs, sugar‐sweetened beverages.

***p < 0.001, **p < 0.01, *p < 0.05, +*p* < 0.10.

Results indicate no significant differences in coercive control and structure across racial and ethnic groups. The only difference was found in the use of autonomy support: Asian fathers reported 0.12 lower scores compared with White fathers and 0.20 lower scores compared with Black fathers. Regarding links between food parenting practices and dietary outcomes, fathers' coercive control was negatively associated with children meeting recommendations for all four dietary outcomes (*b* = −0.41 to −0.27, all *p* < 0.01), while fathers' structure was positively associated with meeting recommendations for all dietary outcomes (*b* = 0.29 to 0.69, all *p* < 0.01). Finally, fathers' autonomy support was positively associated with meeting recommendations for fruit (*b* = 0.20, *p* < 0.05) and vegetables (*b* = 0.41, *p* < 0.001) intake but negatively associated with meeting the recommendation for sugar‐sweetened beverage intake (*b* = −0.17, *p* < 0.05).

Reflecting the limited racial and ethnic differences in food parenting practices, very few significant indirect effects through fathers' food parenting practices were found (Table 5). One significant, albeit small, indirect effect emerged from Asian fathers on children's vegetable intake through autonomy support (*b* = −0.05, *p* < 0.05), which was significantly different from Black and White fathers.

Beyond the very limited indirect connections through food parenting practices, numerous significant differences in children's diets remained across racial and ethnic groups, driven almost entirely by direct effects. Compared with children of White fathers, children of Asian and Black fathers were less likely to meet the recommendation for fruit intake (*b*_Asian_ = −0.28, *p* < 0.05; *b*_Black_ = −0.53, *p* < 0.001). Children of Black fathers were less likely to meet the recommendation for vegetable intake than those of White fathers (*b*_Black_ = −0.39, *p* < 0.05). Children of Black fathers also were less likely to meet the recommendation for fast food intake than children of White and Asian fathers (*b*_White_ = −0.44, *p* < 0.01; *b*_Asian_ = −0.66, *p* < 0.001), while children of Asian fathers were more likely to meet the recommendation than children of White fathers (*b* = 0.24, *p* < 0.05). No statistically significant racial and ethnic differences were found in the direct effects for the likelihood of children meeting the recommendation for sugar‐sweetened beverages intake, although the total effects indicated that children of Black fathers were less likely to meet recommendations than children of Asian fathers (*b* = −0.36, *p* < 0.05). In sum, nearly all of the significant total racial and ethnic differences in children's diets were driven by direct effects, not indirect effects functioning through fathers' food parenting practices.

### 3.3 Moderation Results

A multi‐group analysis was conducted to examine the moderating role of fathers' race and ethnicity in the relation between fathers' food parenting practices and children's diets. The model with all paths constrained fit the data well (*χ* ^2^[92] = 128.758; CFI = 0.971; RMSEA = 0.040; SRMR = 0.060). Furthermore, fit did not significantly differ in comparison to the model in which all main paths were allowed to vary across groups (*χ* ^2^[32] = 68.557; CFI = 0.971; RMSEA = 0.067; SRMR = 0.042), suggesting that associations between fathers' food parenting practices and children's diets did not vary significantly across racial and ethnic groups. The direct associations between fathers' food parenting practices and children's diets identified in the mediation model were replicated in the constrained moderation model (Table 6). In sum, the moderation analyses indicated consistent associations between fathers' food parenting practices and children's diets across racial and ethnic groups.

**TABLE 6:** Moderation Path Model: Multiple Group Analysis by race and ethnicity.

Outcomes and predictors	Hispanic	Asian	Black	White
	*b* (S.E.)	*b* (S.E.)	*b* (S.E.)	*b* (S.E.)
Fruit				
Coercive control	−0.30 ** (0.09)	−0.30 ** (0.09)	−0.30 ** (0.09)	−0.30 ** (0.09)
Structure	0.31 ** (0.12)	0.31 ** (0.12)	0.31 ** (0.12)	0.31 ** (0.12)
Autonomy support	0.19 + (0.10)	0.19 + (0.10)	0.19 + (0.10)	0.19 + (0.10)
Vegetable				
Coercive control	−0.30 ** (0.10)	−0.30 ** (0.10)	−0.30 ** (0.10)	−0.30 ** (0.10)
Structure	0.51 *** (0.13)	0.51 *** (0.13)	0.51 *** (0.13)	0.51 *** (0.13)
Autonomy support	0.42 *** (0.11)	0.42 *** (0.11)	0.42 *** (0.11)	0.42 *** (0.11)
Sugar‐sweetened beverages				
Coercive control	−0.46 *** (0.10)	−0.46 *** (0.10)	−0.46 *** (0.10)	−0.46 *** (0.10)
Structure	0.60 *** (0.12)	0.60 *** (0.12)	0.60 *** (0.12)	0.60 *** (0.12)
Autonomy support	−0.13 (0.10)	−0.13 (0.10)	−0.13 (0.10)	−0.13 (0.10)
Fast food				
Coercive control	−0.33 *** (0.10)	−0.33 *** (0.10)	−0.33 *** (0.10)	−0.33 *** (0.10)
Structure	0.72 *** (0.12)	0.72 *** (0.12)	0.72 *** (0.12)	0.72 *** (0.12)
Autonomy support	0.05 (0.10)	0.05 (0.10)	0.05 (0.10)	0.05 (0.10)

***p < 0.001, **p < 0.01, ^+^ *p* < 0.10.

### 3.4 Alternative Specifications

In order to test the robustness of results, alternative mediation and moderation models were estimated using continuous variables of children's diets (results presented in Tables S1–S5). All dietary outcomes were coded as times per week. As in the results presented above, models using continuous measures of children's intake of fruit, vegetables, sugar‐sweetened beverages, and fast food found that racial and ethnic differences in children's diets were not explained by racial and ethnic differences in fathers' food parenting practices. Similarly, the associations between fathers' food parenting practices and children's diets were consistent across racial and ethnic groups. The model with all paths constrained demonstrated a good fit to the data (*χ* ^2^[92] = 132.030; CFI = 0.952; RMSEA = 0.041; SRMR = 0.050), which was not significantly different compared to the model allowing all main paths to vary across groups (*χ* ^2^[32] = 58.728; CFI = 0.968; RMSEA = 0.057; SRMR = 0.035).

## 4 Discussion

Our study used a sizeable, racially and ethnically diverse sample to examine associations between fathers' race and ethnicity, food parenting practices, and children's fruit, vegetables, sugar‐sweetened beverages, and fast food intake. Replicating prior research (e.g., Hamner 2023), we found substantial racial and ethnic disparities in children's diets, with the predominant pattern showing that children of Black fathers were less likely than their peers to meet dietary guidelines across a range of food types. Also, replicating and expanding past research (Chen et al. 2021), we found that fathers' reported use of structured and autonomy‐supporting food parenting practices was associated with children being more likely to meet dietary guidelines, while their use of coercive control food parenting practices was linked with a lower likelihood of meeting dietary guidelines across all food types. These findings enrich existing literature on fathers of color, which has rarely focused on higher‐order food parenting domains (Parada et al. 2016) and their associations with various dietary outcomes (Lora et al. 2016).

However, importantly, our results did not provide substantive evidence to support either mediation or moderation hypotheses on fathers' race and ethnicity and their food parenting practices. That is, fathers' food parenting practices neither helped to explain racial and ethnic differences in children's diets, nor showed differential associations with children's diets across racial and ethnic groups. These results suggest that factors beyond race and ethnicity, such as family socioeconomic status (SES) and cultural norms, may serve as potential mechanisms driving racial and ethnic disparities in children's diets.

### 4.1 Limited Racial and Ethnic Differences in Fathers' Food Parenting Practices and Connections With Children's Diets

Our primary goal was to assess the role of fathers' food parenting practices in understanding racial and ethnic disparities in children's diets. We tested two conceptual models proposing that (a) dietary racial and ethnic disparities could be driven by racial and ethnic differences in the prevalence of fathers' food parenting practices or that (b) fathers' food parenting practices could be differentially associated with children's diets across racial and ethnic groups. However, neither of these conceptual models received substantive support.

In relation to the first hypothesis, results revealed very minimal differences in fathers' food parenting practices. Across three food parenting domains and four racial and ethnic groups, only one set of significant differences emerged, with Asian fathers reporting lower food parenting autonomy support than White and Black fathers, leading to significant indirect effects (though nonsignificant total effects) on children's vegetable intake. As Asian parents emphasize the consideration of their own opinions over those of their children (Qin and Chang 2012), they may be less inclined to encourage children's independent food choices, which is a key aspect of autonomy support. This lower autonomy support may also reflect traditional Asian cultural expectations regarding fathers serving as the authority figure in the family (Qin and Chang 2012). As such, Asian fathers may be less motivated to promote children's autonomy at the potential cost of losing their authority. Moreover, two items in our study assessing autonomy support require fathers' verbal communication, which may not align with the typical communication patterns of Asian fathers, who have been found to prefer nonverbal expressions of love and support more than verbal communication (Li 2021).

In contrast to this minor pattern of difference in relation to autonomy support, we found that fathers of different racial and ethnic groups showed similar levels of coercive control and structure in their food parenting practices with their young children, replicating limited differences found in prior research (Lora et al. 2016). Other research, however, has identified racial and ethnic differences in food parenting practices among samples comprised primarily of mothers and older children (Polfuss and Frenn (2012)). Together, these results suggest that racial and ethnic disparities in young children's diets may not be due to fathers' differential food parenting practices across racial and ethnic groups. However, additional research is needed to assess whether racial and ethnic differences in fathers' food parenting practices may emerge later in childhood or are more prevalent among mothers than fathers.

Our second hypothesis proposing that fathers' food parenting practices may function differently across racial and ethnic groups also did not receive substantive support, with no significant differences emerging within the moderation analyses. Limited prior research has assessed whether associations between food parenting practices and children's diets vary across racial and ethnic groups, and the few studies revealing racial and ethnic differences in associations focused primarily on mothers. For example, a study by Inhulsen et al. (2017) found that parental (predominantly maternal) controlling food parenting practices were negatively associated with intake of sugar‐sweetened beverages among native Dutch children, but not among nonnative children. In contrast, work by Rahmaty et al. (2023) with an economically advantaged and racially diverse U.S. sample similar to ours suggested that the association between food parenting practices (predominantly among mothers) and preschoolers' BMI did not vary across racial and ethnic groups. Taken together, results from the existing research and our findings suggest that racial and ethnic disparities in children's diets are associated neither with disparities in the patterns of fathers' food parenting practices nor with differences in associations between food parenting practices and children's diets.

### 4.2 Potential Explanations for Children's Dietary Disparities Beyond Race and Ethnicity

Our results highlighted limited racial and ethnic differences in fathers' food parenting practices and their associations with children's diets, patterns that did not explain racial and ethnic disparities in young children's diets. These findings do not contradict arguments within the ecological model of childhood overweight that race and ethnicity play an important role in food parenting and children's diets (Davison and Birch 2001). Rather, our results indicate that when focusing on fathers of color with young children, additional contextual factors not specifically identified in this theoretical model may contribute to the observed racial and ethnic disparities in children's diets. This theoretical inference aligns with broader models of child development (Bronfenbrenner and Morris 2006; García Coll et al. 1996), suggesting that parents' race and ethnicity may not directly affect food parenting practices, but function through broader sociocultural contexts in which families are embedded.

One contextual factor may be family SES: research has consistently noted that children from higher SES backgrounds tend to have healthier dietary patterns than their lower SES peers (Martin et al. 2015). In the U.S., significant variations in SES exist across racial and ethnic groups, with Blacks in particular, as well as Hispanics, experiencing lower SES and higher poverty rates compared to Whites and Asians (Williams et al. 2016), a pattern replicated in the education and income disparities found in our sample. Lower parental income and education may affect children's diets through structural factors related to families' ability to pay for and prepare healthy food options. Due to historical and ongoing racial and economic residential segregation in the U.S., families of color and low SES families are more likely to live in under‐resourced neighborhoods with limited access to healthy food and greater availability of fast‐food restaurants (Richardson et al. 2012). Such barriers may limit families' ability to access healthier food, leading them toward less healthy but more affordable and accessible food choices (Showell et al. 2017).

Beyond economic resources and access to healthy foods, families' cultural beliefs and norms could also contribute to racial and ethnic differences in children's diets. For example, prior studies have highlighted the importance of rice as a staple in the Asian cultural diet (Vue et al. 2011) and the culturally specific cooking methods used in Black families that may involve the addition of extra sugar and fat (Sealy 2010). While these traditional foods may not align with dietary recommendations, minority parents perceived traditional food as crucial for preserving their cultural identities (Roche et al. 2015). More research is needed to investigate further how structural barriers and cultural beliefs are associated with fathers' food parenting practices and children's diets across racial and ethnic groups.

## 5 Limitations

It is important to note several limitations when interpreting these results. First, our study presented correlational results based on cross‐sectional data. Other research designs are needed to investigate causal mechanisms between fathers' food parenting practices and children's diets among fathers of color. Moreover, deriving both food parenting practices and dietary data from a single source may have increased shared error variance, inflating associations between these constructs.

In addition, our measures of food parenting practices and children's diets have several limitations. Although our measures of fathers' food parenting practices were conceptually derived (Vaughn et al. 2016) and empirically validated (Lo et al. 2025), we included limited items with moderate levels of internal reliability in response to fathers' preference for brief surveys. In particular, we only had two items to measure autonomy support, failing to capture other aspects of this construct that may demonstrate racial and ethnic differences. Although the three measures of food parenting practices assessed here have been conceptualized and operationalized across a broad range of research studies, there is a notable lack of consistency in measures, an important challenge for the field (O'Connor et al. 2016).

Moreover, our measures of children's diets were derived from a dietary screener. Although this dietary screener has been validated in prior research (Hewawitharana et al. 2018), it does not capture portion sizes, and hence may under‐ or over‐estimate dietary consumption and does not translate directly into dietary guidelines for children's diet intake (U.S. Department of Agriculture and U.S. Department of Health and Human Services 2020). In addition, since the father is the only reporter of children's dietary intake, reports may not fully account for food that children receive from other sources, such as the other parent, a common challenge in children's nutrition research (Kirkpatrick and Raffoul 2017).

All data in our study were collected through online surveys, which may introduce potential biases related to social desirability in self‐reported data. For instance, fathers may overreport their children's fruit and vegetable consumption or their own use of structure‐based and autonomy‐supportive practices. Such biases are more common in online surveys (Brenner and DeLamater 2016). Additionally, online surveys may be less accessible to families with economic constraints, which could have contributed to the overall economic advantage of our sample. This limits the generalizability of our findings to lower SES families. As discussed earlier, SES may influence fathers' food parenting practices and be a mechanism for explaining racial and ethnic disparities in children's diets. Future research is needed to explore whether patterns observed in our study are found among low SES fathers.

Our sample also has limitations in terms of geographic and racial representativeness. While we recruited fathers from multiple locations to enhance geographic diversity, the sample is not nationally representative. In addition, although we collected data from a diverse group of fathers, sample sizes for Black, Hispanic, and Asian fathers were quite small. This may have limited our statistical power to identify group differences. We also acknowledge the likely variability within the racial and ethnic groups defined in our sample; we were not able to assess potential variability within groups, such as across immigrant and country of origin subgroups. Furthermore, our study excluded fathers from other racial and ethnic groups with limited numbers, such as Native Hawaiian and Pacific Islanders, whose food parenting practices may also differ from the fathers included in the study. Future research would benefit from larger sample sizes of racial minority fathers to explore the intergroup and intragroup diversity of their food parenting practices.

Finally, our study focused exclusively on fathers to address their under‐representation in the food parenting literature (Davison et al. 2020). However, we believe that including co‐parents in research on fathers' food parenting practices could provide a more comprehensive picture of feeding dynamics within families and enhance the robustness of analyses. Given the differences between paternal and maternal food parenting practices (Pratt et al. 2019), further research including both parents in partnered families is essential to examine the distinct contribution of fathers' and mothers' food parenting to children's diets.

## 6 Conclusion

Our study used a sizeable, diverse sample to investigate racial and ethnic disparities in young children's diets and the role of fathers' food parenting practices in understanding such disparities. Results revealed that children of Black fathers were less likely than their peers to meet the recommended intake of fruit, vegetables, sugar‐sweetened beverages, and fast food. Further, results suggested that fathers played an important role in shaping children's diets, with fathers' structure, autonomy support, and coercive control surrounding food all associated with their children's dietary intake. However, these food parenting practices neither mediated nor varied across racial and ethnic groups, suggesting the importance of other factors, perhaps families' sociocultural contexts, in explaining dietary racial and ethnic disparities among young children.

Our findings have implications for reducing racial and ethnic disparities in children's diets. First, given the consistent associations between fathers' food parenting practices and children's diets, results suggest the importance of targeting fathers in early childhood nutrition interventions. It is important for nutrition programs that typically involve mothers, such as the Women, Infants, and Children (WIC) Nutrition Program and Supplemental Nutrition Assistance Program‐Education (SNAP‐Ed), to engage fathers. For example, prior research found that fathers whose partners and children participated in the WIC program expressed a desire to engage with the program to enhance their parenting skills and support their children's nutritional development, yet they faced significant structural and cultural barriers (Dychtwald et al. 2021; Dychtwald and Milliron 2017). In addition, previous interventions focusing on overweight fathers have shown improvement in children's dietary quality (Ashton et al. 2021), indicating the feasibility and effectiveness of such an approach. Including fathers of color in such interventions holds promise for enhancing the dietary quality of racial minority children.

In addition, results finding racial and ethnic disparities in children's diets above and beyond fathers' food parenting practices suggest the need for additional research, as well as associated policies and interventions, tackling the structural and social barriers that prevent children from having healthy diets. For example, food environment interventions and policies are needed to increase access to affordable healthy food, such as incentivizing supermarkets to open in under‐resourced neighborhoods and improving access to healthy food beyond home contexts (e.g., in preschools and restaurants).

## Supporting information


Data S1.

## Data Availability

The data, code, and materials necessary to reproduce the analyses presented here are not publicly accessible. Data, code, and materials are available from the first author (wangecw@bc.edu) upon reasonable request. Analyses were not preregistered.
