# Supplementary material for: Racial and Ethnic Disparities in Fathers' Food Parenting Practices and Children's Diets
Source: Child Dev. 2025 Jun 9;96(5):1718–29. doi: 10.1111/cdev.70001 (PMC12379842; doi:10.1111/cdev.70001)
Supplement: Supplementary file 1 — Data S1. [file CDEV-96-1718-s001.docx]

**Racial and Ethnic Disparities in Fathers’ Food Parenting Practices and Children’s Diets**

**Supplementary Materials**

# Appendix S1

Fathers & Families Study survey questions on fathers’ food parenting practices and children’s diets

***Food Parenting Practices***

The following questions are about your CURRENT food parenting practices, or the strategies you use to feed [child_name]. Please indicate how much you agree or disagree with the following statements when [insert child name here] stays at your home.

1. Do you give this child something to eat or drink if s/he is bored even if you think s/he is not hungry?

- Strongly disagree
- Disagree
- Agree
- Strongly agree

2. Do you give this child something to eat or drink if s/he is upset even if you think s/he is not hungry?

- Strongly disagree
- Disagree
- Agree
- Strongly agree

3. I offer sweets (candy, ice cream, cake, pastries) to my child as a reward for good behavior.

- Strongly disagree
- Disagree
- Agree
- Strongly agree

4. I withhold sweets/dessert from my child in response to bad behavior.

- Strongly disagree
- Disagree
- Agree
- Strongly agree

5. How much do you keep track of the sweets (candy, ice cream, cake, pies, pastries) that your child eats?

- Strongly disagree
- Disagree
- Agree
- Strongly agree

6. How much do you keep track of the snack food (potato chips, Doritos, cheese puffs) that your child eats?

- Strongly disagree
- Disagree
- Agree
- Strongly agree

7. I keep a lot of snack food (potato chips, Doritos, cheese puffs) in my house.

- Strongly disagree
- Disagree
- Agree
- Strongly agree

8. I keep a lot of sweets (candy, ice cream, cake, pies, pastries) in my house.

- Strongly disagree
- Disagree
- Agree
- Strongly agree

9. I try to eat healthy foods in front of my child.

- Strongly disagree
- Disagree
- Agree
- Strongly agree

10. I tell my child that healthy food tastes good.

- Strongly disagree
- Disagree
- Agree
- Strongly agree

11. I encourage my child to eat a variety of foods.

- Strongly disagree
- Disagree
- Agree
- Strongly agree

***Children's Diets***

The following questions are about the different kinds of foods [child_name] ate or drank during the *PAST 30 DAYS*. When answering, please include meals and snacks eaten at home, at school, at restaurants, and anywhere else.

1. During the *PAST 30 DAYS*, how often did [child_name] drink regular soda/pop that contains sugar? Do not include diet soda.

- Never
- Less than once per week
- Once per week
- 2-4 times per week
- Nearly daily or daily
- 2-4 times per day
- 5 or more times per day

1. During the *PAST 30 DAYS*, how often did [child_name] drink sweetened fruit, sports, or energy drinks (such as Kool­Aid, lemonade, Hi-­C, cranberry drink, Gatorade, or Vitamin Water)? Include fruit juices you made at home and added sugar to. Do not include diet drinks or artificially sweetened drinks.

- Never
- Less than once per week
- Once per week
- 2-4 times per week
- Nearly daily or daily
- 2-4 times per day
- 5 or more times per day

1. During the *PAST 30 DAYS*, how often did [child_name] drink flavored milk (such as chocolate vanilla and strawberry milk)? Include flavored milk you made at home. Do not include regular milks or yogurt drinks.

- Never
- Less than once per week
- Once per week
- 2-4 times per week
- Nearly daily or daily
- 2-4 times per day
- 5 or more times per day

1. During the *PAST 30 DAYS*, how often did [child_name] eat fruit? Include fresh, frozen, or canned fruit. Do not include juices.

- Never
- Less than once per week
- Once per week
- 2-4 times per week
- Nearly daily or daily
- 2-4 times per day
- 5 or more times per day

1. During the *PAST 30 DAYS*, how often did [child_name] eat vegetables? Include fresh, frozen, or canned vegetables. Do not include potatoes.

- Never
- Less than once per week
- Once per week
- 2-4 times per week
- Nearly daily or daily
- 2-4 times per day
- 5 or more times per day

1. During the *PAST 30 DAYS*, how often did [child_name] eat something from a fast-food restaurant (such as McDonald’s, Burger King, and Taco Bell)?

- Never
- Less than once per week
- Once per week
- 2-4 times per week
- Nearly daily or daily
- 2-4 times per day
- 5 or more times per day

# Appendix S2

Alternative specifications: descriptive statistics, mediation, and moderation models using continuous measures of children’s fruit, vegetable, sugar-sweetened beverages, and fast food consumption.

## Table S1

*Descriptive Statistics of Children’s Dietary Intake (Times/Week)*

|  | **Full sample**  Mean (SD) | **Asian**  Mean (SD) | **Black**  Mean (SD) | **Hispanic**  Mean (SD) | **White**  Mean (SD) |
| --- | --- | --- | --- | --- | --- |
| Fruit | 13.47 (8.82) | 12.00 (8.92)^w^ | 10.72 (8.82)^h,w^ | 13.92 (9.45)^b^ | 14.12 (8.65)^a,b^ |
| Vegetables | 8.88 (7.64) | 8.96 (7.53)^b^ | 6.72 (8.04)^a,w^ | 7.44 (7.09) | 9.26 (7.61)^b^ |
| SSBs | 2.24 (3.44) | 2.02 (3.18)^b^ | 2.87 (3.58)^a^ | 2.72 (4.03) | 2.17 (3.42) |
| Fast food | 0.71 (0.79) | 0.56 (0.62)^b,w^ | 1.02 (0.97)^a,h,w^ | 0.69 (0.74)^b^ | 0.70 (0.8)^a, b^ |

*Notes.* SSBs: sugar-sweetened beverages. Superscripts indicate significant differences (*p* < .05) from the labeled group: a Asian, b Black, h Hispanic, w White.

## Table S2

*Correlation Between Fathers’ Food Parenting and Children’s Diets*

|  | **Control** | **Structure** | **Support** | **Fruit** | **Vege-tables** | **SSBs** | **Fast food** |
| --- | --- | --- | --- | --- | --- | --- | --- |
| Control | 1.000 |  |  |  |  |  |  |
| Structure | -0.29*** | 1.000 |  |  |  |  |  |
| Support | -0.15*** | 0.39*** | 1.000 |  |  |  |  |
| Fruit | -0.15*** | 0.16*** | 0.13*** | 1.000 |  |  |  |
| Vegetables | -0.18*** | 0.26*** | 0.22*** | 0.43*** | 1.000 |  |  |
| SSBs | 0.13*** | -0.23*** | -0.03 | -0.13*** | -0.12*** | 1.000 |  |
| Fast Food | 0.18*** | -0.26*** | -0.11*** | -0.12*** | -0.14*** | 0.22*** | 1.000 |

*Notes. *** p* < .001. SSBs: sugar-sweetened beverages. Control: Coercive Control. Support: Autonomy support.

## Table S3

*Mediational Path Model Results Linking Fathers’ Race and Ethnicity to Children’s Diets through Food Parenting*

|  | **Linear regression on child diet** | | | | **Linear regression on food parenting** | | |
| --- | --- | --- | --- | --- | --- | --- | --- |
| **Variables** | **Fruit** | **Veg** | **SSBs** | **Fast food** | **Control** | **Structure** | **Support** |
|  | *b* (S.E.) | *b* (S.E.) | *b* (S.E.) | *b* (S.E.) | *b* (S.E.) | *b* (S.E.) | *b* (S.E.) |
| **Father race/ethnicity** ^→^ **Father food parenting** | | | | |  |  |  |
| Asian | | |  |  | 0.01 (0.05) | -0.01  (0.04) | -0.12**^b^ (0.05) |
| Black | | |  |  | 0.04 (0.05) | 0.03  (0.05) | 0.07^a^ (0.05) |
| Hispanic | | |  |  | -0.03 (0.07) | 0.01  (0.06) | -0.01 (0.06) |
| **Father food parenting** ^→^ **child diet** | | | |  |  |  |  |
| Control | -1.76** (0.62) | -1.66** (0.51) | 0.34 (0.20) | 0.17*** (0.05) |  |  |  |
| Structure | 1.92** (0.65) | 3.07*** (0.56) | -1.60*** (0.25) | -0.40*** (0.06) |  |  |  |
| Support | 1.38* (0.59) | 2.24*** (0.47) | 0.47* (0.20) | -0.02 (0.05) |  |  |  |
| **Father race/ethnicity** ^→^ **child diet** | | | |  |  |  |  |
| Asian | -1.48 + (0.78) | 0.12^b^ (0.65) | -0.33 (0.24) | -0.20**^b^ (0.06) |  |  |  |
| Black | -3.11** (0.99) | -2.59** ^a^ (0.89) | 0.27  (0.29) | 0.30**^a, h^ (0.1) |  |  |  |
| Hispanic | -0.28 (1.15) | -1.91* (0.95) | 0.61  (0.53) | 0.00^b^ (0.09) |  |  |  |
| Age | -0.62** (0.21) | -0.04 (0.19) | 0.33 *** (0.07) | 0.06*** (0.02) | 0.05*** (0.01) | -0.01  (0.01) | -0.02+ (0.01) |
| Male | 0.87 (0.53) | 0.03 (0.47) | 0.37+ (0.19) | -0.01 (0.05) | -0.01 (0.03) | -0.01  (0.03) | -0.07* (0.03) |

*Notes.* *** *p* < .001, ***p* < .01, **p* < .05, ^+^ *p* < .10. Veg: Vegetables. SSBs: sugar-sweetened beverages. Control: Coercive Control. Support: Autonomy support. White is the reference group. Significant differences (*p* < .05) between other groups are indicated with superscripts.

## Figure S1

*Mediation Path Analysis Linking Fathers’ Race and Ethnicity to Children’s Diets through Food Parenting*


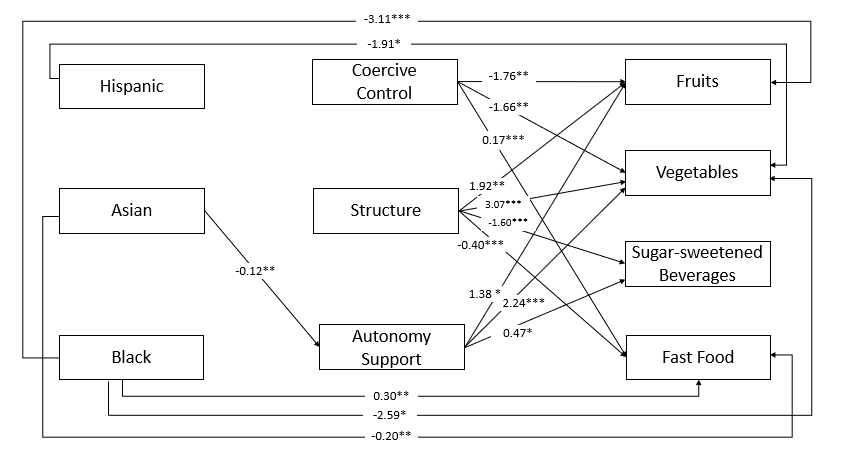


*Notes.* Significant paths (*p* < .05) are presented in the diagram. Unstandardized coefficients are presented for each significant path. Models control for child age and gender. White is the omitted race group.

## Table S4

*Mediational* *Path Model Indirect, Direct, and Total Effects*

| **Indirect effects** | **Fruit** | **Veg** | **SSBs** | **Fast food** |
| --- | --- | --- | --- | --- |
|  | *b* (S.E.) | *b* (S.E.) | *b* (S.E.) | *b* (S.E.) |
| **Asian** |  |  |  |  |
| VIA Control | -0.02 (0.09) | -0.02 (0.08) | 0.00 (0.02) | 0.00 (0.01) |
| VIA Structure | -0.02 (0.08) | -0.02 (0.13) | 0.01 (0.07) | 0.00 (0.02) |
| VIA Support | -0.17+ (0.10) | -0.28*^b^ (0.12) | -0.06+ (0.03) | 0.00 (0.01) |
| Direct effect | -1.48+ (0.78) | 0.12 (0.65) | -0.33 (0.24) | -0.20** (0.06) |
| Total effect | -1.69* (0.77) | -0.20^b^ (0.67) | -0.37 (0.25) | -0.19**^b^ (0.06) |
| **Black** |  |  |  |  |
| VIA Control | -0.07 (0.11) | -0.07 (0.10) | 0.01 (0.02) | 0.01 (0.01) |
| VIA Structure | 0.05 (0.10) | 0.08 (0.15) | -0.04 (0.08) | -0.01 (0.02) |
| VIA Support | 0.10 (0.09) | 0.16^a^ (0.13) | 0.03 (0.03) | 0.00 (0.00) |
| Direct effect | -3.11** (0.99) | -2.59** (0.89) | 0.27 (0.29) | 0.30** (0.10) |
| Total effect | -3.04** (0.99) | -2.42**^a^ (0.89) | 0.28 (0.32) | 0.30**^a,h^ (0.11) |
| **Hispanic** |  |  |  |  |
| VIA Control | 0.06 (0.13) | 0.06 (0.12) | -0.01 (0.03) | -0.01 (0.01) |
| VIA Structure | 0.02 (0.12) | 0.04 (0.18) | -0.02 (0.10) | 0.00 (0.02) |
| VIA Support | -0.01 (0.09) | -0.02 (0.15) | 0.00 (0.03) | 0.00 (0.00) |
| Direct effect | -0.28 (1.15) | -1.91* (0.95) | 0.61 (0.53) | 0.00 (0.09) |
| Total effect | -0.21 (1.18) | -1.84+ (0.97) | 0.57 (0.54) | -0.01^b^ (0.10) |

*Notes.* *** *p* < .001, ***p* < .01, **p* < .05, ^+^ *p* < .10. SSBs: sugar-sweetened beverages. Control: Coercive Control. Support: Autonomy support. White is the reference group. Significant differences (*p* < .05) between other groups are indicated with superscripts.

## Table S5

*Moderation* *Path Model: Multiple Group Analysis by Race and Ethnicity*

| **Outcomes and predictors** | **Asian** | **Black** | **Hispanic** | **White** |
| --- | --- | --- | --- | --- |
|  | *b* (S.E.) | *b* (S.E.) | *b* (S.E.) | *b* (S.E.) |
| ***Fruit*** |  |  |  |  |
| Control | -1.76** (0.58) | -1.76** (0.58) | -1.76** (0.58) | -1.76** (0.58) |
| Structure | 1.92** (0.67) | 1.92** (0.67) | 1.92** (0.67) | 1.92** (0.67) |
| Support | 1.41* (0.59) | 1.41* (0.59) | 1.41* (0.59) | 1.41* (0.59) |
| ***Vegetables*** |  |  |  |  |
| Control | -1.66*** (0.49) | -1.66*** (0.49) | -1.66*** (0.49) | -1.66*** (0.49) |
| Structure | 3.20*** (0.57) | 3.20*** (0.57) | 3.20*** (0.57) | 3.20*** (0.57) |
| Support | 2.24*** (0.50) | 2.24*** (0.50) | 2.24*** (0.50) | 2.24*** (0.50) |
| ***SSBs*** |  |  |  |  |
| Control | 0.35+ (0.19) | 0.35+ (0.19) | 0.35+ (0.19) | 0.35+ (0.19) |
| Structure | -1.60*** (0.22) | -1.60*** (0.22) | -1.60*** (0.22) | -1.60*** (0.22) |
| Support | 0.47* (0.20) | 0.47* (0.20) | 0.47* (0.20) | 0.47* (0.20) |
| ***Fast Food*** |  |  |  |  |
| Control | 0.16** (0.05) | 0.16** (0.05) | 0.16** (0.05) | 0.16** (0.05) |
| Structure | -0.37*** (0.06) | -0.37*** (0.06) | -0.37*** (0.06) | -0.37*** (0.06) |
| Support | -0.03 (0.05) | -0.03 (0.05) | -0.03 (0.05) | -0.03 (0.05) |

*Notes.* *** *p* < .001, ** *p* < .01, **p* < .05, ^+^ *p* < .10. SSBs: sugar-sweetened beverages. Control: Coercive Control. Support: Autonomy support.
